# Early Outcomes of Mental Health Screening Integrated Into Routine HIV Care in Malawi

**DOI:** 10.9745/GHSP-D-23-00517

**Published:** 2024-12-20

**Authors:** Elijah Chikuse, Christine Hagstrom, Deanna Smith, Thokozire Banda, Harrison Chimbaka, Zinaumaleka Nkhoma, Martin Samuko, John Lichenya, Risa Hoffman, Joseph Njala, Sam Phiri, Khumbo Phiri, Joep J. van Oosterhout

**Affiliations:** a Partners in Hope, Lilongwe, Malawi.; b University of California Los Angeles, Department of Medicine, David Geffen School of Medicine, Los Angeles, USA.; c School of Global and Public Health, Kamuzu University of Health Sciences, Lilongwe, Malawi.

## Abstract

Mental health (MH) disorders are highly prevalent among people living with HIV and can have a negative impact on antiretroviral therapy (ART) outcomes. Malawi’s Ministry of Health introduced MH screening in national HIV management guidelines in 2022. We describe early experience with integrated MH screening at ART clinics that have scarce human resources and limited capacity of specialist MH units. ART staff in 15 facilities were trained to use the Patient Health Questionnaire-9 (depression) and the Alcohol Use Disorders Identification Test (harmful alcohol use) screening instruments, MH registers were developed for tracking screening results and referrals, and existing MH referral units were engaged. Based on screening results, ART clients received counseling by lay cadre staff (for mild symptoms) or intensive counseling by trained psychosocial counselors and referrals to specialist MH units (for moderate to severe symptoms). From October 2022 through July 2023, 9,826 ART clients were screened from the following priority groups: returning to care after an interruption in treatment (50%), newly diagnosed (38%), and viral load ≥1,000 copies/mL (12%). Of those screened, 59% were female and 14% were aged 12–19 years. Screening coverage was 85% (9,826/11,553) among the 3 priority groups. All of the individuals who screened positive for moderate/severe depression (1.1%; n=106) or high risk for harmful alcohol use (2.3%; n=227) were referred to specialist MH units. In conclusion, thorough preparation led to high MH screening coverage among ART priority groups, and the number of referrals to specialist MH units was low. MH screening was feasible at Malawi ART clinics. Next steps include studying the clinical impact of integrated MH screening on MH outcomes and ART outcomes (retention in care and viral suppression) and scaling up integrated MH screening to all ART clinics.

## Abstract (precis)

Mental health screening for people living with HIV, conducted mostly by lay cadre health workers, was successfully integrated into antiretroviral therapy clinics in Malawi.

## Teaser

**Key Findings:**

- Mental health (MH) disorders disproportionately impact people living with HIV, affecting their adherence to antiretroviral therapy (ART), care-seeking, and health outcomes.
- At ART clinics in Malawi, lay cadre workers were trained to provide MH screening for depression and harmful alcohol use for clients who were newly diagnosed with HIV, had high viral loads, or were returning to care after treatment interruption and to refer clients to specialized care for MH disorders.
- The vast majority of individuals screened and found with depression symptoms and/or harmful alcohol use could be managed at the ART clinics, with few referrals for more severe cases required.

**Key Implications:**

- Where adequate human and financial resources are available, MH screening should be scaled up to all ART clinics.
- National stakeholders should strengthen the infrastructure at health facilities needed to provide quality, integrated care for MH with sufficient privacy.
- Further implementation research is needed to measure the impact of integrated MH screening on ART and MH outcomes over time and to determine cost-effectiveness.

## BACKGROUND

Mental health (MH) disorders disproportionately impact people living with HIV (PLHIV). Depression prevalence is 2–3 times higher among African PLHIV compared to the general population and is estimated to be between 9% and 32%.^1^ Similarly, harmful alcohol use, defined by the World Health Organization (WHO) as “drinking that causes detrimental health and social consequences for the drinker, the people around the drinker and society at large, as well as patterns of drinking that are associated with increased risk of adverse health outcomes”^2^ is estimated to be more frequent among PLHIV, particularly among men.^2^ Depression and harmful alcohol use increase the risk of acquiring HIV infection and may also result from an HIV diagnosis.^1^^–^^5^ Other psychiatric and anxiety disorders are also highly prevalent among PLHIV globally^4^^,^^5^; 35% of PLHIV in low- and middle-income countries have post-traumatic stress disorder.^6^ PLHIV who experience moderate or severe MH disorders, including depression and harmful alcohol use, are significantly less likely to adhere to antiretroviral therapy (ART), often fail to seek clinical care,^7^^–^^10^ and have increased risk of poor health outcomes, including high rates of viremia.^10^^–^^12^

Successful treatment of MH disorders can improve adherence, viral load suppression, and CD4 recovery among PLHIV^13^; however, MH disorders often go unrecognized in medical care and are, therefore, left untreated.^14^^,^^15^ MH screening in general medical care is insufficient globally. Although there has been a concentrated effort to increase MH screening for PLHIV because of the association between HIV and increased rate of MH disorders, screening is still limited in low- and middle-income countries. This is reflected by the lack of data on the prevalence of depression and harmful alcohol use in the general population and PLHIV in Malawi.^16^^–^^18^

Malawi is a resource-constrained country with an HIV prevalence of 7.1% among people aged 15–49 years.^19^ Malawi’s HIV program has successfully increased ART coverage to 92% and viral load suppression to 85% among adults living with HIV.^20^ While Malawi’s HIV program has been successful in improving HIV outcomes, there is a need for more focus on integrated care to address common comorbidities, including MH disorders. Previously, MH care in Malawi has focused on the management of severe mental illness in the general population. Despite the increased risk of MH disorders, MH care has not been implemented specifically for PLHIV. The need for implementation of sustainable strategies to integrate MH services into HIV care has been recognized globally, with recent guidance on integrated care released by the WHO and Joint United Nations Programme on HIV/AIDS.^21^ Malawi’s Ministry of Health (MOH) responded to this call by introducing MH screening in the national HIV management guidelines in December 2022.^22^ We describe the process of introducing MH screening in an HIV care and treatment program in Malawi, aiming to outline requirements and challenges, including regarding monitoring and evaluation, and draw practical lessons for scaling-up integrated HIV and MH services in similar settings.

The need for implementation of sustainable strategies to integrate MH services into HIV care has been recognized globally.

## INTEGRATING MENTAL HEALTH SCREENING INTO HIV SERVICES IN MALAWI

MH screening was integrated into routine services at 15 ART clinics (7 urban and 8 rural) starting in September 2022 by Partners in Hope (PIH), a Malawian, Christian, nongovernmental medical organization. With funding from the U.S. President’s Emergency Plan for AIDS Relief (PEPFAR) and the U.S. Agency for International Development, PIH supports HIV services at 123 clinics across 9 districts in the country. To implement MH screening at ART clinics, PIH selected screening tools, identified priority populations, trained and prepared staff, piloted and scaled up the program, and conducted ongoing monitoring and evaluation during implementation.

### Screening Tools

**FIGURE 1:**
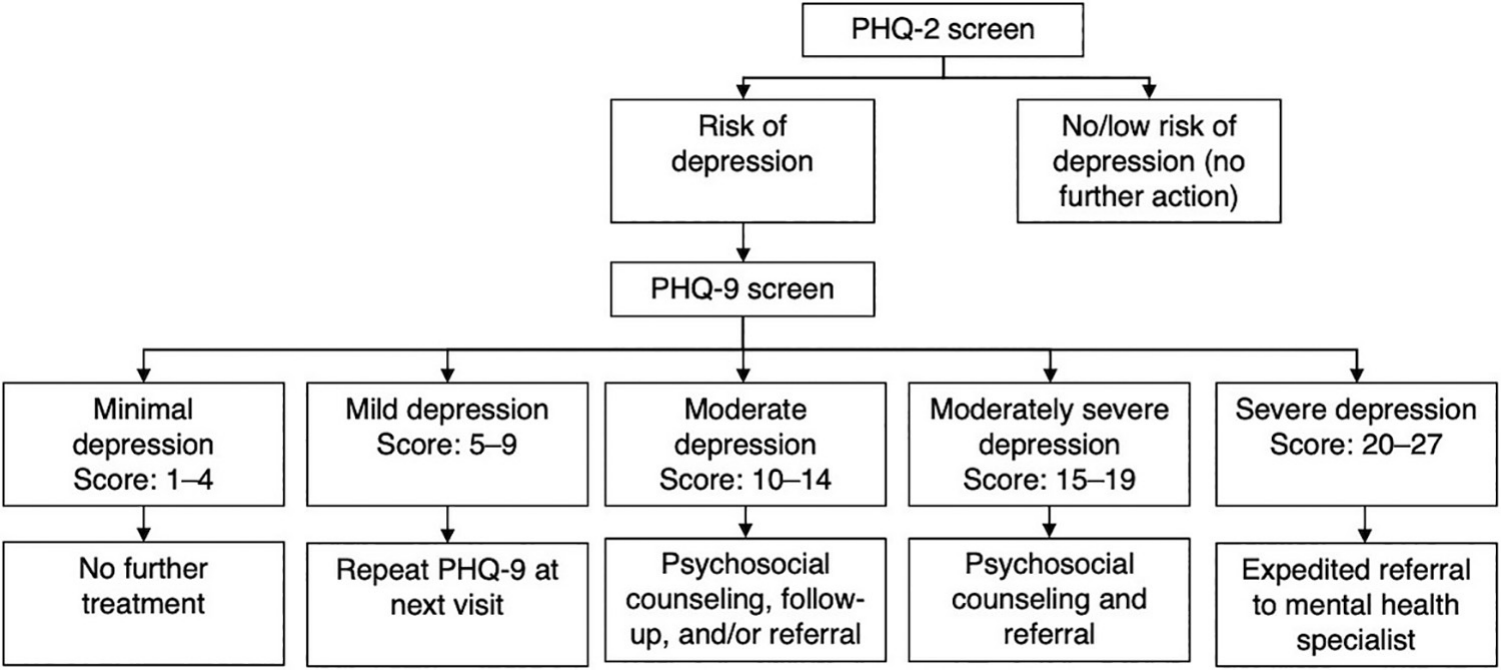
Integrated Depression Screening and Treatment Protocol at ART Clinics, Malawi Abbreviations: ART, antiretroviral therapy; PHQ, Patient Health Questionnaire.

The PIH MH screening focuses on depression and harmful alcohol use, as these conditions have a high prevalence, particularly among PLHIV.^1^^,^^3^ Feasible, evidence-based screening tools and interventions for both conditions are available.^23^^,^^24^ To screen for depression, we used the Patient Health Questionnaire (PHQ)-2 followed by the PHQ-9 tool, as it has been validated in Malawi and used in HIV clinics in sub-Saharan Africa.^24^^,^^25^ The PHQ-2 is a preliminary tool to screen for depression and asks clients how often they’ve experienced “little interest or pleasure in doing things” and “feeling down, depressed, or hopeless” in the last 2 weeks. If a client answers yes to either of those 2 questions, the remaining 7 more detailed questions of the full PHQ-9 tool, which diagnoses and measures the severity of symptoms, are subsequently asked.^26^ Clients with high scores (15 or more), indicating severe symptoms, were referred to MH specialists based on Malawi’s MOH guidelines’ proposed treatment actions (Figure 1).^22^^,^^27^

**FIGURE 2:**
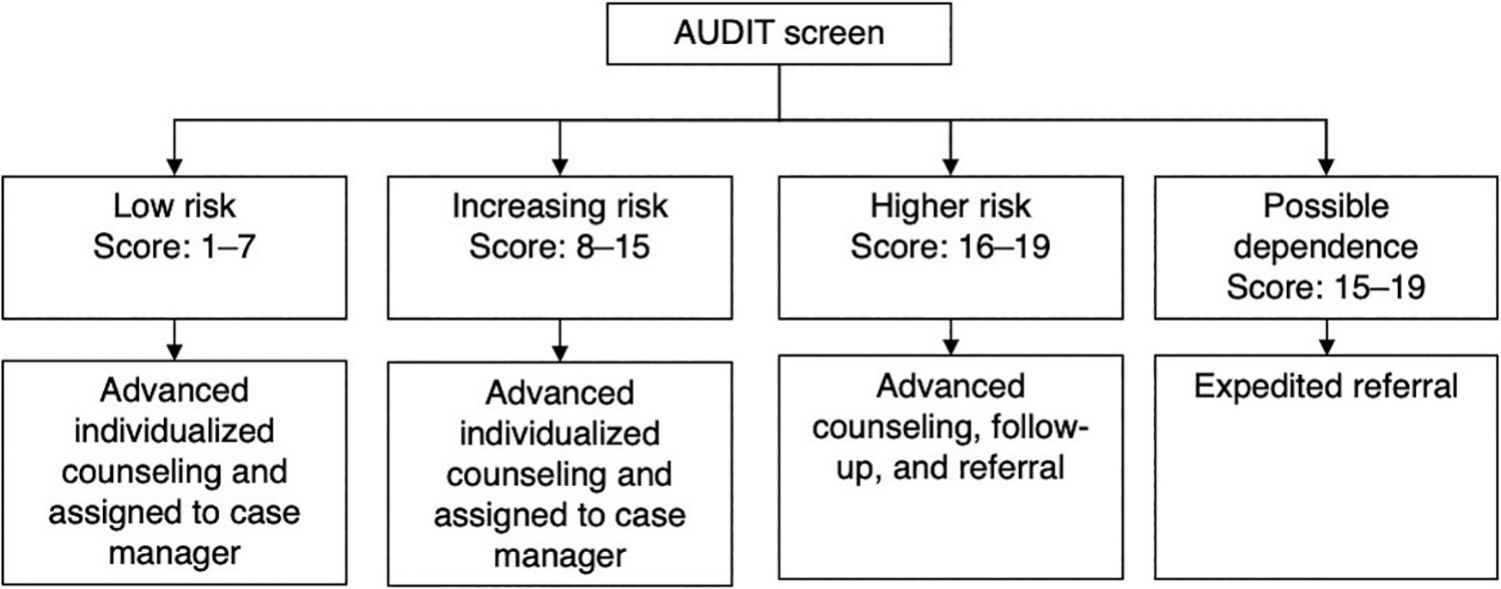
Integrated Harmful Alcohol Use Screening and Treatment Protocol at ART Clinics, Malawi Abbreviations: ART, antiretroviral therapy; AUDIT, Alcohol Use Disorders Identification Test.

To screen for harmful alcohol use, the Alcohol Use Disorders Identification Tool (AUDIT) was selected because it has been previously used among PLHIV in Malawi with high acceptability and fidelity.^28^ The AUDIT tool is a 10-question screening tool developed by the WHO to screen for problematic alcohol use.^29^ Using the AUDIT manual’s proposed treatment actions as a guide,^30^ clients with scores of 16 or more were referred to MH specialists for further treatment (Figure 2).

### Priority Screening Population

MH screening focused on “priority clients,” defined as people newly diagnosed with HIV, people with high viral load results (≥1,000 copies/ml), and clients who have experienced treatment interruption and were returning to care. In line with national guidelines,^22^ only priority clients were screened due to their presumed higher risk and because screening all ART clients was not feasible with the available human resources. Priority clients are actively identified at the clinics, newly HIV-diagnosed clients are identified at testing sites, clients with high viral load are identified at ART clinics, and clients who have missed appointments or experienced attrition from care are identified through clinician review of individual treatment charts and health passports. These specific priority groups already received a standard package of care, including adherence counseling, psychosocial counseling, and health education, focused on their status of new HIV diagnosis, high viral load, or interruption in treatment. MH screening was added to the standard operating procedure for this standard care package.

### Training and Preparation

Treatment supporters, HIV diagnostic assistants, and psychosocial counselors were selected to conduct MH screening. Treatment supporters and HIV diagnostic assistants are lay cadre workers who support clients receiving HIV care. Lay cadre workers were selected to conduct MH screening based on the task-shifting model from the WHO^31^ and evidence demonstrating the effectiveness of task-shifted MH care.^32^

Treatment supporters conduct health education, adherence counseling, treatment literacy education, navigation through integrated care, and tracing of clients with treatment interruptions. At a minimum, they have a high school certificate and a 5-day dedicated training on HIV and psychosocial counseling.

HIV diagnostic assistants screen and identify clients in need of HIV testing and assist newly diagnosed PLHIV with enrollment into care. They hold a high school certificate and complete a 4-week training on HIV testing services.

Psychosocial counselors are professional counselors who are trained in health sciences and have, at a minimum, a diploma in psychosocial counseling. They managed clients with moderate depression symptoms and clients in the increasing risk and higher risk AUDIT score categories who required advanced psychosocial counseling. They also mentored treatment supporters and HIV diagnostic assistants in MH screening and counseling.

Most clients were screened by treatment supporters, HIV diagnostic assistants, or psychosocial counselors, but occasionally, this was done by a clinician or nurse, who was also responsible for follow-up of any medication side effects, HIV treatment adherence, ART outcomes, and quality of life. Of note, no additional lay cadre or professional staff were employed to conduct MH screening. This task was added to their existing responsibilities, as described earlier, without changes to the organizational structure at the health facility level.

PIH trained 138 treatment supporters, HIV diagnostic assistants, and psychosocial counselors for 3 pilot sites and 235 additional staff for the scale-up to 12 additional sites. An overview of depression and harmful alcohol use among PLHIV is already part of the 5-day training that is conducted for all lay cadre workers, which prepares staff to assess and manage clients experiencing MH challenges and refer them to appropriate services. The specific 1-day training for integrated MH screening included how to use the PHQ 2/9 and AUDIT screening tools with practical examples and the processes for documentation, data reporting, and referral. After the training, regular supportive supervision took place, focused on the fidelity with which implementation of MH screening, management, and referral took place. This supportive supervision took place monthly, lasted around 2 hours, and was integrated into regular mentoring for HIV services to strengthen the overall competencies of lay cadre staff. Staff at clinics where MH screening was introduced but who were not directly involved in it received a 2-hour orientation.

The specific 1-day training for integrated MH screening included how to use the PHQ 2/9 and AUDIT screening tools with practical examples and the processes for documentation, data reporting, and referral.

Before implementation, PIH mapped all existing MH service delivery points in the districts and engaged with specialist MH clinics to be prepared for referral of severe cases from our integrated MH-ART screening sites.

### Pilot and Scale-Up

Screening was piloted from February to September 2022 in 3 high burden (5,000–7,000 clients on ART) facilities in 3 different districts. The decision to begin with a limited number of clinics was part of our original program work plan, based on availability of resources and training funds. Because the pilot screening and referral process proved feasible without notable challenges, scale-up to 12 more high-burden facilities took place in October 2022. High-volume facilities were purposively selected for MH screening across geographical regions of Malawi.

### Implementation

Screening was conducted in a counseling room at the clinics, although privacy was not always possible, given the limited space available. The PHQ 2/9 and AUDIT tools were read aloud to clients. Based on screening results, ART clients with mild MH symptoms received counseling from treatment supporters and HIV diagnostic assistants. ART clients with moderate to severe disorders (PHQ-9 score of 15 or more and/or AUDIT score of 16 or more) received intensive counseling from psychosocial counselors and referrals to professional clinical officers, medical doctors, and nurses trained in MH services at existing specialist MH units. Depending on the severity and prognosis, clients had monthly or biweekly MH management sessions with assessments at every follow-up visit to monitor progress on treatment. Two district hospitals in our program (Chikwawa and Mulanje District Hospitals) have MH referral units on-site. On-site referrals were made through documentation in the client’s health passport. Clients at all other integrated MH screening ART clinics who required referral had to travel to an MH referral unit. Off-site referrals followed routine referral procedures by which clients were told which day to go, based on knowledge of opening times of MH referral units, but individual appointments were not made.

### Monitoring and Evaluation

The screening was conducted using paper forms and attached to individual client ART charts. Screening results and referrals were also documented in a custom-made register. Data from paper registers were entered into a database, with data summaries and analyses displayed in a dashboard, which was generated monthly and shared with staff at the health facility, district, and central levels. Monthly trends of screening outcomes and screening coverage by priority group, health facility, and district level were reviewed by district and central teams to inform programmatic implementation, such as identifying ART clinics requiring additional supervision, mentorship, and support for MH screening. Interventions were followed up at quarterly review meetings for district staff.

Quality assurance during implementation was conducted by randomly selecting cases from the MH screening register and cross-checking with the screening results attached to the client’s chart. Further checks were done by comparing the MH screening scores with documentation in psychosocial counseling forms.

## FINDINGS

**FIGURE 3:**
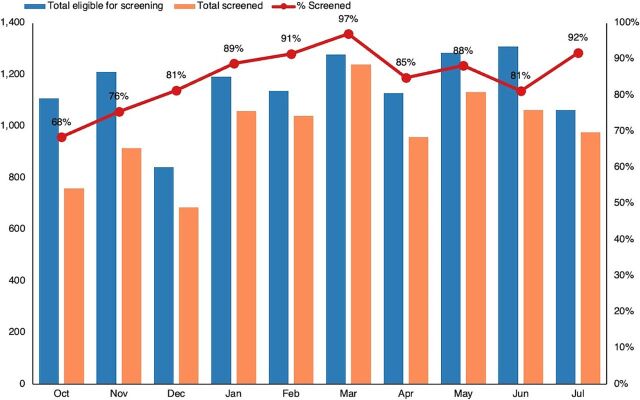
Mental Health Screening Coverage at 15 High-Burden ART Clinics, Malawi, October 2022–July 2023 Abbreviation: ART, antiretroviral therapy.

From October 2022 through July 2023, there were 11,553 eligible for screening among the 3 priority groups. Of these, 9,826 ART clients were screened from these mutually exclusive client categories: returning to care after treatment interruption (50%), newly diagnosed (38%), and high viral load (12%). The screening coverage rate was 85%, with moderate monthly variability after the first 2 months (Figure 3). MH screening coverage was the highest among clients with high viral load (87%), followed by clients returning to care (86%) and newly diagnosed (83%). The reason for the somewhat lower screening coverage among newly diagnosed is likely that some were linked to care on the weekends when staff were not always available for MH screening.

More female (59%) than male clients were screened, reflecting the female predominance of the ART client population. The majority of clients screened were aged 35 years and older (52%), followed by 20–34 years (36%) and 12–19 years (12%).

**FIGURE 4:**
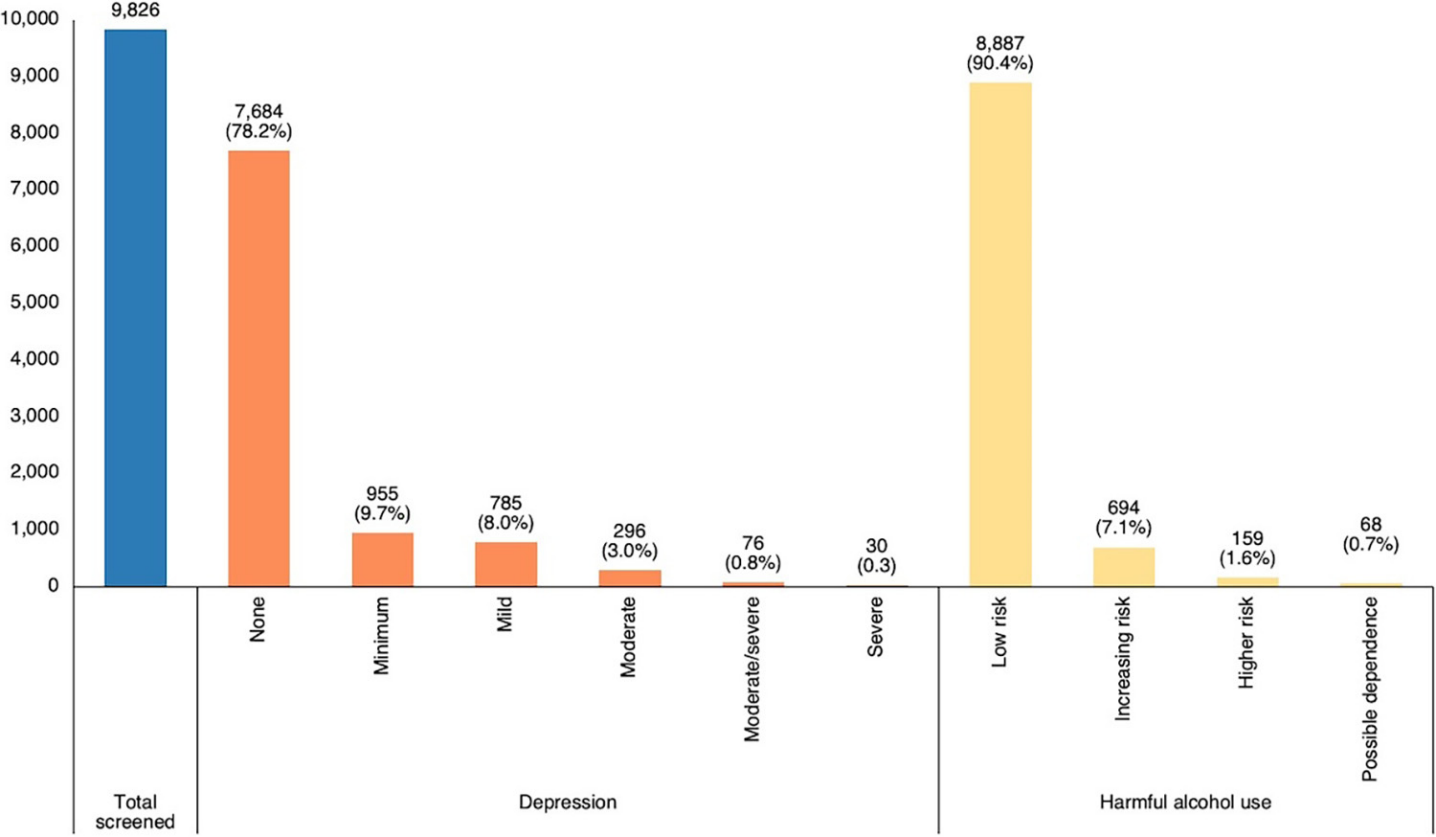
Categorized Depression^a^ and Harmful Alcohol Use^b^ Screening Results at 15 High-Burden ART Clinics, Malawi, October 2022–July 2023 Abbreviation: ART, antiretroviral therapy. ^a^Patient Health Questionnaire-9 depression scores: none=0, minimum=1–4, mild=5–9, moderate=10–14, moderate/severe=15–19, severe=20–27. ^b^Alcohol Use Disorders Identification Test for harmful alcohol use scores: low risk=1–7, increasing risk=8-15, higher risk=16–19, possible dependence≥20.

The majority of clients did not screen positive for depression (7,684; 78.2%) and had a low risk of harmful alcohol use (8,887; 90.4%) (Figure 4). A total of 106 clients (1.1%) had PHQ-9 scores of more than 15 and 227 (2.3%) had high-risk AUDIT scores. All 333 individuals were referred to MH units.

Of the 9,826 clients screened, 78% did not screen positive for depression and 90% had a low risk of harmful alcohol use.

## DISCUSSION

This is the first documentation of an integrated HIV-MH service in a programmatic setting in Malawi that has been evaluated with relevant, high-quality data. In our setting, MH screening and referral, conducted mainly by lay cadre health workers, was feasible at high-burden ART clinics given that high coverage of MH screening (85%) among selected priority groups was achieved over time and that all eligible, high-risk clients with severe symptoms were referred to specialist MH units. We recognize that high-quality integration of MH screening among ART priority groups was in part possible due to availability of human resources in the context of PEPFAR support at these 15 sites. Other facilitating factors were the strong collaboration between staff from PIH and MOH at health facilities and the mentoring and supportive supervision of lay cadre staff by experienced PIH district teams that included clinicians, nurses, and psychosocial counselors.

The number of clients in severe categories of depression and harmful alcohol use was low compared to several previous programs.^33^^–^^35^ The reasons for this include differences in the screening tools used, categorization of screening outcomes, and populations screened. However, it could also suggest that the screening quality by lay cadre staff was inadequate, as further discussed later. The capacity to provide advanced psychosocial counseling and the burden on MH referral units may be more challenging in other settings where rates of severe depression and harmful alcohol use are higher. Integrated MH screening placed an additional burden on available lay cadre staff conducting the screening, counseling, and data entry. This is relevant given the increasing number of health services that are recommended to be integrated into HIV services^22^ (hypertension, diabetes, family planning, and cervical cancer), requiring effort from the same lay cadre staff.

### Challenges and Limitations

While our program demonstrates the feasibility of implementing screening for depression and harmful alcohol use within specific contexts, there were also challenges and limitations. First, we were not able to screen all priority clients. Temporary declines in screening coverage were noted when trained lay cadre staff were replaced by colleagues who had not yet been trained in MH screening. As we did not record the duration of these interruptions of availability of trained staff, we could not determine the magnitude of the effects.

Secondly, we do not have comparison data to determine whether screening results would differ had it been conducted by more advanced health professionals. Although our approach of providing lay cadres with training, supervision, and mentorship has been demonstrated to lead to adequate MH screening in Malawi,^22^ inadequate skills among some staff may have affected screening quality and need further exploration.

Another challenge we faced was a lack of privacy due to inadequate infrastructure at many health facilities. We attempted to conduct screening with sufficient privacy, but private spaces were often unavailable. Because of the stigma associated with MH disorders, clients may have avoided answering questions honestly if they were concerned that responses could be overheard by other clients.

A further limitation is that we did not capture prospective cohort data due to our reliance on routinely collected, aggregated program data and did not triangulate paper-based MH screening data with ART outcomes and subsequent viral load results in electronic monitoring record systems. Due to often incomplete documentation of outcomes of MH referrals in clients’ health passports, we could not consistently track whether clients attended their referral appointments, what their treatment consisted of at the MH referral units, nor their MH outcomes. Additionally, there is anecdotal evidence that not all clients who were referred reached off-site MH units, potentially due to lack of transportation or personal reasons, such as stigma. Adapting the HIV program’s electronic monitoring record system may be the best solution for more advanced monitoring of MH screening and management (including for referrals), but this requires significant effort and can only take place at the national level.

Shortage of MH specialists,^37^ shortage of antidepressant medication,^38^ and lack of capacity among providers at referral sites may have also hindered clients from receiving additional support. In this MH screening initiative, financial input costs were mainly for human resources for health (effort dedicated to MH screening), training, and mentoring/supervision, while limited expenditure was incurred for administrative supplies (registers and screening forms). These input costs were nearly fully covered by PIH’s PEPFAR grant for HIV care and treatment, drawing attention to the sustainability of integrated HIV-MH services in Malawi. Previous initiatives that initially successfully integrated MH screening into HIV care found sustainability a notable challenge. For example, a pilot program demonstrated the successful introduction of depression screening and treatment in Malawi, but screening did not continue when program funding ended, primarily due to human resources and infrastructure challenges.^22^^,^^35^^,^^36^

## RECOMMENDATIONS

Based on our program’s success with integrating MH screening into ART clinics and the challenges identified, we suggest scaling up integrated MH screening in ART clinics with the following recommendations.

- Despite funding restrictions and health system challenges, integration of MH services into HIV programs should have high priority, given the repercussions of MH disorders for HIV treatment outcomes and the fact that successful, integrated MH screening with basic management and referral is feasible with limited investments.
- Regular refresher trainings about the MH integration process and screening tools should be held to sustain and improve screening coverage over time. In addition, there is some research demonstrating successful task-shifting with MH screening,^32^ but further studies may be needed to test whether the quality of MH screening by lay cadre workers matches that of health care professionals.
- A tracking system should be implemented to improve feedback to clinics/providers about the outcome of MH referrals. When implementing an integrated screening program, we recommend including a comprehensive, bidirectional referral strategy and feedback system between the screening and referral sites.
- For comprehensive monitoring and evaluation of integrated MH services, and in particular, for associating MH screening results and outcomes with ART outcomes, a more advanced data collection and management system should be available. There are opportunities to include integrated MH services as a module in the ART electronic monitoring record system, similar to what is already present for hypertension. This would also make cohort analysis and analysis of individual client outcomes feasible.
- Integrated MH screening at ART clinics takes place in a health system that is insufficiently equipped for high-quality MH services.^39^ National efforts are needed to improve general MH care by qualified MH staff, enhance privacy at health facilities, facilitate access to specialized MH care, and provide a consistent medication supply.

## CONCLUSION

In conclusion, after thorough preparation, we achieved high MH screening coverage among ART priority groups. The number of referrals of cases with severe symptoms was low in relation to the number screened, which may have limited the extra burden on specialist MH units. MH screening was feasible in our setting at Malawi ART clinics. Our next steps will include studying the clinical impact of integrated MH screening on MH outcomes and ART outcomes (in particular, retention in care and viral load suppression) and scaling up integrated MH screening to all ART clinics.

## Notes

**Peer Reviewed**

**First Published Online:** November 22, 2024.
